# “We have become doctors for ourselves”: motives for malaria self-care among adults in southeastern Tanzania

**DOI:** 10.1186/1475-2875-13-249

**Published:** 2014-07-02

**Authors:** Emmy Metta, Hinke Haisma, Flora Kessy, Inge Hutter, Ajay Bailey

**Affiliations:** 1 Ifakara Health Institute, PO Box 78373, Dar es Salaam, Tanzania; 2 Population Research Center, Faculty of Spatial Sciences, University of Groningen, Landleven 1, 9742 AK Groningen, The Netherlands; 3 Mzumbe University, PO Box 20226, Dar es Salaam, Tanzania

**Keywords:** Malaria, Self-care, Adults, Qualitative methods, Self-medication, Tanzania

## Abstract

**Background:**

Prompt and appropriate treatment of malaria with effective medicines remains necessary if malaria control goals are to be achieved. The theoretical concepts from self-care and the health belief model were used to examine the motivations for malaria self-care among the adult population.

**Methods:**

A qualitative study was conducted through eight focus group discussions with adult community members to explore their general opinions, views and perceptions of malaria and of its treatments. These groups were followed by 15 in-depth interviews of participants with a recent malaria experience to allow for an in-depth exploration of their self-care practices. The analysis followed principles of grounded theory and was conducted using Nvivo 9 qualitative data management software.

**Results:**

The self-treatment of malaria at home was found to be a common practice among the study participants. The majority of the participants practiced self-medication with a painkiller as an initial response. The persistence and the worsening of the disease symptoms prompted participants to consider other self-care options. Perceptions that many malaria symptoms are suggestive of other conditions motivated participants to self-refer for malaria test. The accessibility of private laboratory facilities and drug shops motivated their use for malaria tests and for obtaining anti-malarial medicines, respectively. Self-treatment with anti-malarial monotherapy was common, motivated by their perceived effectiveness and availability. The perceived barriers to using the recommended combination treatment, artemether-lumefantrine, were related to the possible side-effects and to uncertainty about their effectiveness, and these doubts motivated some participants to consider self-medication with local herbs. Several factors were mentioned as motivating people for self-care practices. These included poor patient provider relationship, unavailability of medicine and the costs associated with accessing treatments from the health facilities.

**Conclusions:**

Malaria self-care and self-treatment with anti-malarial monotherapy are common among adults, and are motivated by both individual characteristics and the limitations of the existing health care facilities. There is a need for public health interventions to take into account community perceptions and cultural schemas on malaria self-care practices.

## Background

Globally, malaria remains a menace. An estimated 219 million cases are reported annually worldwide leading to 660,000 deaths, with sub-Saharan Africa (SSA) accounting for 90 percent of the cases [1]. Malaria is an important cause of death in SSA [1] where 75 percent of 650 million people reside in malaria transmission areas. In Tcbuanzania 10–12 million clinical malarial cases are reported annually by public health facilities, with the disease contributing to more than 40 percent of visits to health facilities [2]. A recent disease-specific mortality estimate estimates the cumulative probability of dying from malaria in Tanzania at 81.1 per 1,000 population [3]. Under-five children and pregnant women are the most susceptible to malaria transmission and its subsequent effects. Although the overall malaria burden is generally decreasing throughout Africa [4-9], a shift in the burden to adults is reported [10]. Thus, in this paper the focus is on adults and their motives for malaria self-care practices.

As malaria is preventable and curable [1,5] large-scale anti-malaria interventions have been initiated by the Roll Back Malaria Partnership (RBM), the Global Fund to Fight AIDS, Tuberculosis and Malaria and the President’s Malaria Initiative (PMI). Currently, four main malaria control interventions are being pursued: improved malaria case management, intermittent malaria treatment in pregnancy, malaria epidemic prevention and control, and integrated malaria vector control activities. These interventions are aimed to reduce the incidence of malaria by at least 75 percent and to nearly eliminate the number of malaria-related deaths by 2015 [11]. The scaling-up of these interventions has already led to a decline in the malaria burden in areas where the disease is endemic [1,12].

Consequently, the World Health Organization (WHO) recommends prompt and appropriate management of all malaria cases with efficacious medicines [1]. Most SSA countries have started to use arteminisinin combination therapy (ACT), which is efficacious in the management of uncomplicated malaria cases. In Tanzania, a fixed-dose combination of artemether-lumefantrine (ALu) has been used since 2006 for treating such uncomplicated malaria cases. The use of ACT replaced sulphadoxine/pyrimethamine (SP) that was hitherto used for treating uncomplicated malaria cases on children above two months of age and or weighing above 5 kgs and all adults except in last trimester pregnancy cases, and lactating mothers whose babies are below two months of age [13,14]. To enhance access to ACT, ALu is also made available at subsidized prices in the private retail sector, which is dominated by accredited drug dispensing outlets (ADDO) in Tanzania. The ADDO initiative is aimed at improving the quality of care in the private drug retail sector [15]. Other anti-malarial drugs, particularly monotherapy, are also readily available in these outlets.

People in different communities may have different opinions about illnesses, which inform their schemas and perceptions of the efficacy of various medical options. Indeed, cultural norms and beliefs tend to shape treatment-seeking practices [16-18], including perceptions about susceptibility to malaria, the consequences of the disease and the effectiveness of various malaria treatments [19-21]. Self-medication for malaria using retail drugs has been found to be common [22], in SSA in general [23-27] and Tanzania in particular [28-31]. Thus, in this paper, the focus is to understand the malaria self-care practices and motives for the choice of drug store services.

### Self-care as a concept

The concept of self-care in public health can be traced back to the 1960s [32]. It has been defined as a process in which individuals undertake disease prevention, detection and treatment on their own without consultation to healthcare providers; it entails self-diagnosis and use of remedies previously prescribed for similar illness and/or the purchase of medications without professional advice [32-38]. Segall and Goldstein describe self-care as comprising potential behavioural practices such as health maintenance, disease prevention, assessment of symptoms, self-diagnosis, self-treatment (non-medication treatments and self-medication), self-referral, consultation with non-medical (alternative) care practitioners, and the use of specialized medical care [34]. WHO acknowledges self-care as a key aspect of public health, which plays a pivotal role in the health care system [22], despite the attendant risks, including those associated with incorrect diagnosis and use of non-prescribed medicines [39].

In public health research, theories and models on health-seeking behaviour help explain the underlying motivations for often multifaceted and complex people’s care-seeking decisions [40]. In particular, the health belief model (HBM) is widely applied to explain health-seeking behaviour. This model centres on four concepts: the *perceived susceptibility* to a disease, the *perceived severity* of the disease, the *perceived benefits* of the treatment actions, and the *perceived barriers* to adopting proposed measures. The model posits that what triggers individuals’ decisions regarding health-seeking behaviour are general health motivations, including internal cues (i.e. symptoms), and external cues (i.e. interpersonal interactions and media communications) [41]. Subsequently, the concept of *self*-*efficacy* was added to the model to refer to an individual’s perceived ability to adopt and perform specific actions successfully. This individual-centred model is aimed at assessing the decision-making processes employed by individuals in need of healthcare.

The HBM concepts are also pertinent to self-care decisions. In this regard, an individual’s perceptions of his or her susceptibility to malaria and the perceived severity of the condition will inform his/her perceptions of the threats associated with leaving the condition unattended to. This study has combined the theoretical concepts from Self-care and HBM, to argue that self-care practices are more likely to be informed by individuals’ knowledge of malaria and perceived susceptibility to the condition. Moreover, individuals’ perceptions regarding the severity of the disease and the perceived benefits associated with different treatments options for restoring health will inform their self-care therapy choices.

### Self-care in Tanzania

Studies on health care-seeking behaviour in response to malaria among Tanzanians [42-46] shows that adults reportedly turn to professional healthcare only when the symptoms persist or worsen [43,44,46]. Research suggests, however, that the medicines bought in drug stores are often of poor quality, and are not used rationally in many developing countries [47], including Tanzania [28,48,49]. Concerns that most malaria self-treatment practices are inadequate, inappropriate and ineffective have been expressed in relation to Tanzania [29,30] and elsewhere [25,50,51]. In fact, self-care increases the chances of using unnecessary medicines while delaying seeking of appropriate treatment of the illness, which may exacerbate and complicate the disease. In this regard, the role of self-care in the development of drug resistance, especially to anti-malarial drugs, cannot be underestimated [23,24,50-53]. In this paper, the specific attention is on why people choose self-care practices in malaria treatment, and the motives for the choice of drug store services. Gaining such an understanding of the motives behind malaria self-care is critical in developing strategies for promoting the use of appropriate medicines in the community. This study was conducted as part of a larger project that investigated health-seeking behaviour among adults in the context of the epidemiological transition, with a focus on malaria and diabetes. The results from other parts of the project will be presented separately.

## Methods

### Study area and population

This study was conducted in Kilombero, one of the six districts of the Morogoro region, located in the southeastern part of Tanzania. Kilombero district has a total area of 14,018 sq km and a population of 407,880 (202,789 men and 205,091 women) [54]. There are 54 health facilities in the district, including two hospitals (a designated district referral hospital and a hospital owned by a private organization), four government health centres and 48 dispensaries, some of which are owned by the government, and some of which are privately owned. The Kilombero district has long been known for its malaria intensity [55,56], and although the incidence of malaria has been reduced, the disease is still the leading cause of morbidity and mortality in the district [8]. The districts’ ecological and climatic condition is suitable for both high and perennial malaria transmission [57]. The malaria control programmes of the district have benefited for more than 50 years from the malaria research activities of the Ifakara Health Institutes, as well as from the efforts of several other malaria control stakeholders in the district.

Two villages were purposively selected for the study with the help of district council health management team members. The criteria for the section based on their proximity to the designated district hospital. The semi-urban village was located less than 5 km from Ifakara town, while the distance between the rural village and the town was 43 km. This discrepancy provided the opportunity to capture semi-urban and rural perspectives regarding self-care practices. The total population of the two villages is 21,270: 12,823 people live in the semi-urban village and 8,447 people live in the rural village. The majorities of the villagers have no more than a primary school education, and are small-scale farmers who grow mainly rice and maize. The main sources of health services for the residents of the semi-urban village are a laboratory facility and four dispensaries, all of which are privately owned, and which diagnose and treat malaria, along with other health problems. There are also 14 drug shops located in the village that sell a variety of medicines, including anti-malarial drugs. The village has no government-owned health facilities. As they live close to Ifakara, the residents of this village can also access a number of drug shops, laboratory facilities and dispensaries located there. By contrast, the rural village has one private laboratory facility that provides diagnostic services only, and a government-owned village dispensary. This dispensary provides both diagnostic tests and treatments for malaria and other conditions. The inhabitants of this village can obtain anti-malarial drugs from the four drug shops located in their village or from several other shops in the adjacent villages. They also have the option of travelling to Ifakara to access treatments or purchase medicines. There is no privately owned health facility in this village.

### Study design and participant recruitment

This is a qualitative, exploratory study in which the views of adults were examined through focus group discussions (FGDs) and in-depth interviews (IDIs) conducted between October 2012 and March 2013. The purpose of the FGDs was to elicit a broad range of views and opinions on malaria, malaria self-care practices and motives for utilization of drug shop services. The goal of the IDIs was to explore in greater depth individual perceptions and experiences with malaria, and the decision-making processes involved in malaria self-care practices. The age range of the participants was 25–81 years. Of the 74 study participants (35 women and 39 men), 59 were involved in eight FGDs (with each made up of seven to nine participants), and 15 participated in the IDIs. In order to capture a wide range of opinions and ideas, and taking into account the gender dynamics in the community, FGDs were held separately for men and women. The number of discussions and interviews was determined after data saturation had been reached. The FGD participants were purposively selected among the adult residents of the community. The FGDs participants’ inclusion criteria considered gender, being an adult and a permanent residency in the study villages.

IDIs were conducted with adult individuals who had direct experience with malaria 14 days prior to the date of the interview. These individuals were purposively selected from the community with the help of the village leaders and none of them were involved in the FGDs. In determining eligibility for participation in the study, the research team visited individuals in their villages, asking them about their fever history over the previous two weeks, and using screening questions to determine the individuals’ beliefs about the causes of the fever. After it was established that malaria was the cause of an individual’s fever, and that the individual met the study’s inclusion criteria, the person was given detailed information about the study, and was asked whether he/she was willing to take part. If the individual expressed an interest in and a willingness to participate, appointments for discussion were made based on his/her availability. Most of the discussions took place two days after the appointments at the participant’s household.

### Data collection

Two research assistants with a social science background at postgraduate level were trained to conduct the study. Together with authors, these assistants piloted the data collection guides. The results from the pilot helped the team to refine the guides before they were used in the main study. The data collection took place in two separate rounds. The first round involved FGDs. The results from this round were used to sharpen the IDI topic guides before they were used in the second round of data collection, which consisted of the IDIs. All of the FGDs and the IDIs were facilitated by the authors. In each of the FGDs, one of the assistants took notes. Both FGDs and IDIs were conducted in Swahili, a language which was spoken by a majority of the participants. These discussions were recorded, and verbatim transcriptions of the recordings were undertaken within 48 hours of the time they were conducted. The transcripts were reviewed and cross-checked for quality by the authors before they were imported to NVivo 9 (QSR International Pty Ltd, Australia). All the transcripts were analysed in their original language. The analysis process took place at two levels. The first level entailed the development of inductive and deductive codes. This was followed by a second level, in which the codes were categorized into themes and family codes based on the principles of the grounded theory and the concepts of the HBM. After the deductive (HBM) and the inductive concepts were combined, the following themes emerged: (i) knowledge and malaria awareness; (ii) malaria self-care practices; (iii) self-diagnosis and use of pain killers (iv) self-diagnosis and the prescription of anti-malarial drugs (v) self-referral for a malaria test (vi) self-medication: a preference for monotherapy; (vii) motives for using drug shop services; and, (viii) self-medication with alternative medicines.

### Ethical approval

The study was given ethical approval by the institutional review boards of the Faculty of Spatial Sciences, the University of Groningen in The Netherlands, the Ifakara Health Institute (IHI) in Tanzania, and the National Tanzanian Medical Research Coordinating Committee of the National Institute for Medical Research (NIMR). Study participants in both FGDs and IDIs provided verbal consent. To ensure the anonymity of the study participants, all identifiers were removed from the data, and only the opinions of the participants are presented.

## Results

### Knowledge and malaria awareness

Levels of knowledge and awareness of malaria shape people’s perceptions regarding their susceptibility to the disease, and are key factors in health care-seeking practices. In this study, the majority of the participants displayed a basic knowledge of malaria, including the possible consequences of the disease and its link with mosquitoes. In the FGDs, participants described malaria as a major health problem in the community and as a threat to the lives of the majority of people. In general, the participants agreed that everyone in the community was potentially susceptible to the condition, regardless of his or her age. The high levels of knowledge and awareness of malaria observed in this setting reflect the prevention efforts of the malaria control organizations in the Kilombero Valley. These organizations conduct intensive media campaigns on malaria, which include road shows, posters and leaflets. Recently, radio and television have been used for malaria control education, albeit mainly in urban and semi-urban areas. However, radio, and especially the local radio station *Radio Pambazuko Kilombero*, is a key source of malaria information in rural areas. The following explanation of how media raises community awareness of malaria and its link to mosquito was offered in an IDI:

“*Yeah*, *it is what we are told everyday mosquitoes are the ones causing even in the radios*, *hospital there*…*there are advertisements*/*posters on mosquitoes causing malaria*… *every day you will hear mosquito is a dangerous insect*… *it causes malaria*… *malaria is dangerous present quickly in the hospital to get service when you think you have malaria*”. Ashura female, 53 years^a, b^.

In all of the discussions, malaria, or *homa ya malaria*, was said to produce a wide range of symptoms, including high fever, coldness, tiredness, headaches, yellow vomit, dizziness, joint pain, loss of appetite, yellow urine, and diarrhoea. These symptoms are in line with clinical descriptions of the disease. However, the participants observed that these symptoms are not always taken seriously as signs of malaria due to their perceived non-specificity. During the interviews, most of the participants said they did not become convinced that the symptoms were suggestive of malaria until after they had used a painkiller and found that the symptoms persisted. Participants considered a malaria test to be important in confirming their self-diagnosis, due to similarity of symptoms to typhoid and other diseases.

### Malaria self-care practices

In this study, malaria care seeking is seen as a process that entails different activities at different levels. The participants expressed concerns about the cost of using health care facilities, frequent shortages of medicines, and the attitudes and practices of health care workers; noting that factors such as these affect their health-seeking behaviour and discourage them from using the services of health care facilities. In outlining their perceived malaria symptoms, the participants reported several self-care strategies (see Table 1) based on their evaluation of the severity of their symptoms. They also considered their perceptions regarding their ability to use self-care strategies successful, and their perceptions of the benefits of employing such strategies. A close analysis of the data revealed that the care-seeking behaviours of the participants were similar in both the semi-urban village and the rural village, and among men and women. There were no apparent gender differences in the self-care practices, though affordability was more of a concern among women than men. In elaborating on the malaria self-care practices identified in this study, the following sections are structured according to the theoretical concept of Segall and Goldstein [34] explained earlier in the paper.

**Table 1:** **In**-**depth interview participants**’ **self**-**care actions to their recent malaria episode**

**Name**	**Sex**	**Age (years)**	**First action taken**		**Second action taken**			
			**Self-medicate/use painkiller**	Use AM*	**Use AM**	**Nothing**	**Laboratory diagnosis**	**Visit health facility**
Uzuri	Female	26	Yes					Yes
Zed	Male	26	Yes				Yes	
John	Male	37	Yes			Yes		
Salome	Female	66		Yes				
Xemp	Male	46	Yes				Yes	
Easter	Female	34		Yes				
Gift	Female	51	Yes				Yes	
Kheri	Female	60	Yes				Yes	
Mchome	Male	50	Yes		Yes			
Barua	Male	65	Yes		Yes			
Ashura	Female	53	Yes		Yes			
Daima	Male	65	Yes				Yes	
Kapera	Male	60	Yes				Yes	
Mfuko	Male	46	Yes					Yes
Cheupe	Female	34	Yes				Yes	

*AM: Anti-malarial medicine.

### Self-diagnosis and use of painkillers

A majority of study participants reported engaging in self-diagnosis and self-medication of fever and pain using painkillers, such as Panadol® (paracetamol) (see Table 1). Participants perceived that the symptoms resulted from normal tiredness due to hard work or working in the sun, and that the painkiller would help. The perceived benefits of the painkiller in reducing fever and alleviating pains were also cited as motives for self-medication. Most of the painkillers used were either leftovers of medication used to treat previous illnesses, or they were bought from general shops. The participants explained that when the painkiller did not provide them with relief from their symptoms, they then started to consider further options.

“… *after using that Panadol I did not get big changes*, *I was getting little relief and after sometimes that condition relapses into more severe form that is why I thought it can be malaria and true when I went for a to test I found it is malaria then I used medicines*”. Salome, female, 66 years.

The study participants reported waiting two to three days after they first experienced the symptoms before taking further action. The decision to wait was based on the expectation that the symptoms would subside after the use of the painkiller. It was not until participants perceived their symptoms as more severe, which also increased their perception of susceptibility to malaria, that they reported taking further action.

“*It took like two to three days* … *because I was thinking it is just a normal tiredness due to work but after observing I have used Panadol with no success it is when I thought I better go for testing so that I know for sure what exactly is the problem*”. Xemp, male, 46 years.

The general use of painkillers observed in the study may have been shaped by an individual’s long history of treating febrile conditions, and his or her perceptions of the benefits of painkillers in alleviating fever and pain. The long-term impact of such practices on the health of a user is of concern, but addressing this issue was not possible in the current study.

### Self-diagnosis and the prescription of anti-malarial drugs

A majority of the participants had been residents of the study communities for many years. As malaria has been endemic in the region, the people have learned to live with it by developing cultural schemas for self-care. This could be seen in the IDIs, in which some participants reported self-medicating with anti-malarial drugs as soon as they had symptoms, based on their strong perception that malaria was the cause. Participants described their decision-making process regarding the use of anti-malarials as follows:

“*When I had that fever I knew completely it is malaria due to how I was feeling* … *that cold*, *headache and joint pains all these were severely paining*, *I knew it will only be malaria and nothing else*… *so I decided to use anti malaria and I am doing well*”. Salome, female, 66 years.

Individuals’ previous experiences in managing similar conditions seem to have informed their self-medication decisions. There appears to be a large reservoir of medical knowledge in the community on malaria and its treatments. This might be due to the social marketing interventions in the area, coupled with individuals’ multiple exposure to bouts of the disease and to its treatments. Thus, self-diagnosis and self-prescription were often reported by the study participants:

“… *so after feeling that condition*… *I thought it is better I buy that amodiaquine because it is a medicine that I am used to and whenever I have a malaria condition like now* ..*when I just use it my condition becomes better. I could have bought other medicines but there was no need because my medicine which I am used to was available*”. Mchome, male, 50 years.

### Self-referral for a malaria test

The perceived severity of symptoms and the increased susceptibility to malaria triggered the need for a test. Majority of IDI participants indicated they obtained malaria tests from private laboratory diagnostic facilities. Only a few of the interviewees went to a health care facility for a laboratory test after painkillers failed to relieve their pain. These private laboratory facilities are found within the catchment area of the study communities, and are managed by trained laboratory practitioners equipped to diagnosis conditions such as malaria using a blood sample, and examination of other illnesses using urine and stool samples. At the time of this study, most of these facilities were using microscopic diagnostic tests for malaria that cost 0.64$ (Tshs 1,000). The interviewees reported referring themselves to these facilities to confirm their self-diagnosis before deciding on which medicine to use.

The perceptions that some malaria symptoms resemble those associated with a urinary tract infection (UTI) or typhoid were among the reasons cited for choosing to take a malaria test. The participants observed that self-diagnosis could lead to the wrong choice of medications, leaving the true cause of the condition untreated, and thus prolonging the duration of the illness:

“…*It does happen even though not to myself but you may hear someone has taken malaria medicines even for three times but the fever do not stop*… *when they go for checking* [*testing*] *they find the person has typhoid while he was treating malaria*… *that is why he was not getting cured so a situation like that does exist and that is why it is important to test first before taking any medicines to be sure which medicines you use for what disease*…” Gift, female, 51 years.

The high levels of awareness of malaria and of testing options found in this study can be attributable to the behaviour change and communication (BCC) activities regarding prompt and effective malaria treatments that have been undertaken in the district. Some of the participants made reference to the instructions and information people receive from different media channels on malaria treatment. The following statements describe in detail some of the cues and motives for taking a malaria test:

“*Because information is already going around*… *people are being instructed*…. *the media as well*… *is saying* ‘…*people you should not just start taking medications before testing to know what disease is disturbing because sometimes you may be suffering believing it is malaria while it is not*….*it is just another disease* … *now when you use anti*-*malaria medicines you cannot get cured as a result you just hurt yourself*’... *so we listen to them that is why we test first for malaria before using anti malaria medicines*… *we want to know if it is malaria*, *how many parasites are there and if it is not malaria what is the problem that disturbing*…” Daima, male, 65 years.

### Self-medication: a preference for monotherapy

Despite the changes in malaria treatment guidelines in Tanzania that recommend the use of combination therapy, other anti-malarial treatment, many monotherapy, are widely accessible through the private sector. The results of this study show that a variety of anti-malarial medicines were used in managing participants’ recent malaria episodes. The use of monotherapy was reported by the majority, leaving less than half of the participants using the combination treatment/ALu that is recommended as the first-line anti-malarial in Tanzania. However, a number of participants reported using a variety of monotherapies in managing their recent bouts of illness, including Metakelfin®, Fansidar®, Malafin®, Orodar® and amodiaquine. These medicines were obtained from drug shops. Most of the interview participants reported choosing anti-malarial brands after the drug shop clerk or service provider asked them: “*What anti*-*malarial medicine do you normally use*?”.

The perceived benefits and effectiveness associated with specific medicines prompted their choice. The individuals’ previous experience with using the same medication to manage similar conditions may have motivated their decision:

“…*that one*.... *I just decided myself to use Orodar because that is the medicine I normally use when I get malaria*… *if I use that Orodar it does not disturb me I just become well and continue with my normal activities*”. Ashura, female, 53 years.

A number of participants reasoned the length of the treatments and the need to take multiple tablets as some of the barriers for using ALu therapy. In elaborating on this, participants gave examples in which they compared the ease of using some monotherapy, especially single-dose, with using ALu, which must be taken over three days:

“…*the problem with Mseto*/*ALu is those tablets* … *they have made so many tablets* …*when the patient sees them* …*he gets scared* ….*because I don*’*t know they are like how many*…. *24 tablets something like that*… *and you are supposed to take all of them*… *that also I think is not good*… *because when one is sick first of all you just don*’*t like medicines*.... *now when the dosage has many tablets it particularly disappoints you*… *I think it is better they reduce the number of tablets a bit*… *it will easy its use* (will you use it?) *no honestly I like the one to use only once*, *these one* [ALu] *I think*… *I will fail completely*… *I will just ask the doctor to prescribe me another one to use once*… *isn*’*t it that there are just various malaria medicines*….” Uzuri, female, 26 years.

However, the quality of medicines purchased in drug shops, and specifically the monotherapy may be questionable, and their use may prevent malaria sufferers from using more effective combination treatments.

### Motives for using drug-shop services

Drug shops play a major role in managing malaria illness in the community. In this study, all of the participants except one said they had obtained their anti-malarial medicines from drug shops, regardless of where the diagnosis was made. These shops are widespread and easily accessible in the study villages. The privately owned shops offer various types of anti-malarial drugs, usually with or without a prescription. The majority of the clerks in these shops are government trainees certified to sell a set of essential drugs, including the combination treatment/ALu.

When asked why they chose to use drug shops, many participants cited the cost of going to health care facilities. During group discussions, participants reported that when they think of visiting health care facilities, many negative associations came to mind. For example, one participant said a person would have to ask himself/herself: “*Do I have enough money to get treatments there*?” before visiting such a facility. This concern was explained in detail in the interviews, in which participants reported not seeking care from the health facilities because of limited funds:

“…*due to the financial position one has*, *one may have 500 shillings only at hand*, *when you go there at the health facility you are asked 1*,*000 shillings for registration right*… *you are not yet given medication*… *you are told to go and buy from the drug shop*… *now it is better I take a short cut to buy medicines from the drug shop and use*… *it is not that we fail to go for testing there* … *it is a result of the financial ability*….” Barua, male, 65 years.

Participants reported taking such shortcuts to obtaining medical services through providers such as drug shops, because they knew visiting health care facilities would entail more expense, i.e. for consultation, testing and medicines, which they believed they could not afford:

“ ….*as a result of the amount of money I had*… *I thought if I go there at the dispensary it won*’*t be enough to buy medicines because I have to pay for consultation first*… *that is why I decided taking short cut to buy medications straight away and use*”. Easter, female, 34 years.

Members of the discussion groups complained that in most cases the health care facilities do not have malaria medicines on the premises, and that when they visit such a facility they have to pay for consultations with a practitioner who provides them with a paper prescription, which they then have to take to the shop to be filled. Participants voiced their concerns that drug shortages, coupled with the procedures patients have to follow at the health care facilities, forces patients to perform the double role of both patient and doctor. Thus, the study participants reported that taking responsibility for one’s own diagnosis, prescription, outcome evaluation, and re-diagnosis is the norm. Health care facilities are seen as a last resort when self-care options have failed.

“…*to a big percentage*… *we have become doctors for ourselves because we are tired of going to the health facilities. When one feels headache*, *leg pain*, *high fever*… *you will hear saying go and buy me Mseto*/*Alu*… *he buys it and use*, *if he still feels fever he goes to buy tablets for* … *for Amoeba believing that may be this is Amoeba*, *when he uses it and find still not yet* …*will go to maybe to his*/*her neighbour* …*the neighbour will tell him perhaps it is urinary truck infection* [*UTI*] *so he will go to the drug shop*, *buy medicines and use. Now after seeing he*/*she have used all these medicines and the condition is still persisting* … *it is when another person will come and tell him maybe it is typhoid*, *so now with typhoid he*/*she will go for testing*… *currently people are tired of going to the health facilities*… *we have become doctors ourselves because drug shops are many and sometimes when you go to the drug shop they advise* … *use this medicine* … *so you feel it is better I go to the shop rather than wasting all my time going to the health facility*, *pay for consultation and waste the a full day there at the end being told to go to buy medicines from the shop*…*isn*’*t it better going to the shop directly*?” Female FGD1, 25 – 51 years, semi-urban village.

The participants also reported that the attitude toward patients of the typical health care provider was among their motivations for seeking care from the shops. They said they believed they were not getting enough attention during health facility consultations, or that they had to wait to receive services:

“*Others* [*doctors*] *when you get there he just asks you one question*…‘*what is the problem*’…*now as you continue explaining your problem just at the middle of the explanation he gives you a book* [*a prescription*] *saying* ‘*go and buy these medicines*…’ *you see*…*now this medicine I am going to buy is for what*? *I haven*’*t yet explained my problem well for him to hear now he has the prescription already* … *then when a person sees that*… *would he really go to the health facility again*? *You will go to the health facility to do what*? *Then it is when you think you better go to the drug shop and explain to that seller s*/*he would advise*”. Female FGD4, 25 – 46 years, rural village.

### Self-medication with alternative medicines

Self-medication using alternative medicines was mentioned in both the FGDs and in IDIs as representing a common practice in the study communities, although none of the participants indicated that they had used such medicines in managing their recent malaria episodes. Alternative medicines, such as neem tree roots and leaves (*mwarobaini*), were frequently mentioned as being used for treating malaria in the community. Among the alternative medications mentioned were aloe vera and mlonge (*Moringe oliferus*) leaves. It is widely believed that the use of local herbs can significantly delay the recurrence of malaria. The participants cited the perceived benefits and efficacy of the herbs as motivation for their use as self-medication by the majority of people in the community:

“… *it is just many* [*people*] *who use local herbs and if you use the tree* [*Neem tree*] *you stay for a very long time* … *can be even six months before getting malaria fever again*… *that is real a medicine as what the elders just said* … *that tree is very helpful* …” Male FGD2, 43 – 80 years, semi-urban village.

The perceived benefits related to availability and self-efficacy of local herbs was also cited as reasons why they are used instead of western medicines. In many cases, local herbs can be accessed at zero cost, as people can just pick leaves or roots from the specific trees available in the vicinity. The perceived benefits that local herb do not have side-effects was cited as another motive for their use. In addition, participants noted that many people doubt the efficacy of the recommended treatments for malaria, and thus prefer to use local herbs. The presence of various anti-malarial drug brands on the market and the changes in anti-malarial treatment policies were cited as signs of the government’s uncertainty regarding the effectiveness of anti-malarial medicines, and were thus seen as cues for favouring the use of local herbs:

“*I think there are two things*… *first treatment costs* …*if you don*’*t have money what will you go for at the health facility*?… *you will not get any help*… *secondly the government is not sure of what is doing*… *as it is making changes on malaria treatment medicines from time to time*… *this becomes like they are not yet certain on what medicine to use* … *instead they are deciding to do research on us*… *so who wants to be researched on*? *That is why people do not go there* [*the health facility*]… *they don*’*t like those medicines they believe it is better to use local medicines because first of all*…. *these local medicines do not have side effects* ….” Mfuko, male, 46 years.

Two of the interview participants reported that they often self-medicate using herbal remedies when they feel unwell, even though they did not do so in the most recent episode. A number of participants expressed the belief that herbal medicines are preferable to conventional medicines when a disease is still in its early stages. They stated that alternative medicines act more slowly on the human body than western medicines, and are thus more effective in managing mild conditions:

“....*what caused me not to use local herbs* …*is that local herbs treat slowly*… *I mean*… *One should not wait until a person becomes completely serious then you start giving him*/*her local herbs*… *that is not right because at that stage you cannot give the medication since local herb treatments delays but these medicines of our colleagues*… *western medicines* …*treats quicker. For instance if a person is dehydrated you cannot be saying bring him local herbs and start administering*….*you will welcome death. So my condition was a bit worrying and had not yet prepared the medications* … *I thought I use first these western medicines then I see how it goes*”. Xemp, male, 46 years.

Several participants also said that alternative medicines could be useful in preventing diseases in some instances. The Swahili term *mwarobaini* translates as ‘of forties’. This ‘forties’ concept is reflected in the widely held perception that *mwarobaini* can treat more than 40 diseases. As a consequence, many community members believe that when this medicine is used, other health problems that may be present in the body will also be eliminated:

“…*for us who use this mwarobain we know that it treats many diseases*… *so you can use it* ….*if there is another health problem in the body then it clears as well. Sometimes you may find maybe rashes coming out from the body* ….*so when you have rashes in the body* …*there is another tree that you can use*….*and that mwarobain you can as well take*, *boil and bath with*…*all the rashes will disappear*”. Kapera, male, 60 years.

Despite their use in managing malaria in the community, the long-term impact of alternative medications on the health of the users is not clear, and might need further exploration.

## Discussion

Successful malaria treatment depends largely on early recognition of the disease symptoms and the subsequent treatment through the use of effective anti-malarial medicines. The current study reveals that care seeking is a complex process characterized by multiple layers of actions. The perceived susceptibility to the disease and the meaning people attach to their symptom experiences underlie many of the actions taken. Self-medication with painkillers leftover from the treatment of previous illnesses or bought from drug shops as an initial response to illness was found to be common. Perceptions regarding the mildness of the symptoms and the perceived benefits of using a painkiller to relieve the condition motivated the decision to self-medicate. Individuals decided whether to seek additional help after observing the progress of the illness, and their decisions were motivated by their perceptions of the severity of the condition and of their susceptibility to the disease. The higher awareness on malaria seen in the study could be a possible explanation for the observed consistency in self-care practices among men and women. The current study focused on malaria self-care practices among adults. The results are comparable to those of the studies conducted in other parts of Tanzania [46,49] and other malaria endemic regions [25,58,59] where self-medication was the first response employed to malaria. This practice is unsafe as it may delay effective treatment and prolong the sufferer’s illness. The failure to seek prompt and effective treatment within 24 hours of the onset of malaria symptoms can prevent communities from reaching malaria control goals set by the government and global malaria control organizations.

The correct diagnosis of malaria is indispensable in ensuring that malaria sufferers are treated appropriately. The government, in line with WHO’s recommendations [60], advises people who suspect they have malaria to take a malaria test before receiving treatment, in the form of either microscopy or a rapid diagnostic test. The results of this study showed, however, that the majority of people wait to take anti-malarial drugs until after they have taken a malaria test to confirm their self-diagnosed condition. The failure of the initial self-medication practices to produce relief, the perceived severity of the symptoms and the perceived degree of susceptibility to the condition motivated the study participants to take a malaria test. A preference for testing before taking anti-malarial drugs has also been reported in other settings [18,42]. Although a malaria test before the use of anti-malarial drugs is recommended [60], the quality of the malaria test services offered, especially in the private sector, is questionable, and the use of such tests can lead to the over-diagnosis of malaria [61]. The over-diagnosis of malaria has considerable implications for malaria control, as it triggers the unnecessary use of anti-malarial drugs, which can increase the chances of developing drug resistance [62], and can exacerbate the suffering that comes from untreated illnesses. In light of the preference for a malaria test expressed in the study, and of the need to safeguard the efficacy of anti-malarial medicines, as the recent WHO’s T3: Test Treat Track initiative for scaling up diagnostic tests indicated, it may prove necessary to implement quality controls and follow-up surveillance of malaria treatments.

Despite the shift to ACT in the country’s malaria treatment policy, self-medication with monotherapy was found to be common among study participants. The participants expressed a preference for sulphadoxine-pyrimethamine, sulphamethoxypyrazine-pyrimethamine and amodiaquine medicines over the recommended combination treatments/ALu. Individual participants’ experiences in managing a similar condition and their perceptions of the benefits and effectiveness of specific brands strengthened their confidence in their self-treatment choice. These perceptions were often reinforced by the ease of using monotherapy, which are administered as single doses. The continued use of monotherapy, despite the recommendation that combination treatments be used, has also been reported in Ghana, where chloroquine, artemether, amodiaquine, quinine, and sulphadoxine-pyrimethamine are used by the majority of malaria sufferers [59]. The ongoing use of these brands for malaria management might seriously impair malaria control efforts, as anti-malarial monotherapy is known to cause resistance to malaria parasites [27,62-64]. Thus, their use may contribute to the increased resistance of malaria parasites to effective anti-malarial medicines.

Recent reviews [23,27] have shown that drug shops are the main source of malaria medicines used for self-treatment. The same observation was made in the current study. The cost of medication and services, shortages of medicines, waiting times for receiving services, and the attitudes toward patients displayed by health care workers were cited by participants as motives for accessing drug shops directly. These findings are comparable to results previously reported for the research district [56,65] and for other settings [66]. Although many people obtain medicines from shops when self-medicating for malaria [28,30,31,49,56,67], the quality of those medicines is worrying [48,68,69]. The use of ineffective treatments can lead to avoidable morbidities and deaths, and to the continuing spread of disease parasites in the community. To improve the quality of the medications dispensed in drug shops, the ADDO programme was implemented in Tanzania in 2006 [15]. The continuous monitoring of the quality of the medicines sold in drug shops, and of dispensing behaviour in these shops, would be beneficial. The availability and accessibility of more effective anti-malarial drugs, such as ACT, in drug shops need to be assessed. In addition, community members should be informed of the potential benefits of ACT, and encouraged to use them.

The use of local herbs in self-treatment of malaria was reported as a normal practice in this study. The participants indicated that they perceive local herbs, or *mwarobaini*, as having no negative effects on the user and as being effective in curing multiple conditions. Although the use of local herbs for malaria management is not new in Tanzania [19,21,70,71] or in other parts of SSA [72-74], the motives for the practice appear to be changing. Previous studies have found that many people O;JKLassociate severe malaria with *degedege*, which may lead them to choose traditional/local medicines [16,18,19,21,70,75]. The results of this study indicated that local herbs are perceived as being effective in treating mild conditions. A lack of faith or uncertainty regarding the effectiveness of the recommended combination treatments/ALu was among the reasons cited by participants for the use of local herbs. The availability of various anti-malarial drug brands in the market, coupled with frequent policy changes regarding treatments, has led many members of the community to question the efficacy of the recommended treatments. However, it is not clear whether the use of local herbs can lead to a concentration of toxins that could be harmful to the human body. Currently, little is known about the ability of local *mwarobaini* herbs to treat malaria, or about their safety in humans.

This study was conducted in a district with a long history of malaria disease, including communities that have been intensively involved in several malaria control and management interventions [68,76]. The finding that self-care is practiced widely in this particular setting suggests it is also practiced elsewhere. However, as the study was conducted using a small group of people in a specific setting, the results should be viewed as contextual, and any generalization of the findings should be limited to this kind of setting. Nevertheless, the study’s results provide valuable insights into the motives for malaria self-care among adults in the community. This is important, as it can help to inform policy decisions regarding the planning and implementation of programmes aimed at improving the effective and appropriate management of malaria.

## Conclusion

This study has shown that malaria self-care among adults is motivated by people’s perceptions and schemas on the disease and the health care options available to them. The actions taken by the study participants reflect the economic inequalities that exist in the community. Though there have been sustained malaria interventions in the region the access to appropriate treatment has been limited. There is a need for public health interventions to take into account community perceptions and cultural schemas on malaria self-care practices.

### Endnotes

^a^All quotes have been translated from Swahili to English hence they may not follow strict grammatical rules.

^b^All names used are fictitious, and do not reveal the true identity of the study participants.

## Abbreviations

SSA: Sub-Saharan Africa; PMI: President’s Malaria Initiative; RBM: Roll Back Malaria; ACT: Artemisinin combination therapy; ALu: Artemether-lumefantrine; ADDO: Accredited drug dispensing outlets; HBM: Health belief model; FGD: Focus group discussion; IDI: In-depth interviews; WHO: World Health Organization; NIMR: National Institute for Medical Research; UTI: Urinary truck infection; BCC: Behaviour change communication; NMCP: National malaria control programme.

## Competing interests

The authors declare that they have no competing interests.

## Authors’ contributions

EM conceived and designed the study, collected the data, performed the analysis and the interpretation of the data, and wrote the manuscript. FK oversaw the data collection process and contributed to the interpretation of data and to the drafting and editing of the manuscript. HH and AB conceived the study and oversaw all aspects of the study, including drafting and editing of the manuscript. IH conceived the study, provided technical support in the study design and in the editing of the manuscript. All of the authors read and approved the final manuscript.
